# DISPARITIES IN HUMAN CAPITAL INVESTMENT OVER THE GENDERED LIFE COURSE: AN INTERNATIONAL COMPARISON

**DOI:** 10.1093/geroni/igz038.009

**Published:** 2019-11-08

**Authors:** Phyllis Cummins, Takashi Yamashita, Christopher Phillipson

**Affiliations:** 1 Scripps Gerontology Center, Miami University, Oxford, Ohio, United States; 2 University of Maryland, Baltimore County, Baltimore, Maryland, United States; 3 The University of Manchester, Manchester, United Kingdom

## Abstract

Income disparities by gender are a persistent problem throughout the world. These disparities place women at risk for economic insecurity both while working and in retirement. Education and continued skill upgrading are key to reducing income disparities, but it is well documented that both older men and women are less likely to participate in adult education and training (AET) than their younger counterparts. In this symposium we present gender and age-based differences in AET in Australia, Canada, England/Northern Ireland and the United States. Also, given the increasing use of technology, technology-related problem-solving skills are compared across these four nations. In addition, we discuss current, and potentially new, country level policies and practices that facilitate the provision of AET over the second half of the life course. Yamashita and colleagues use data from the Program for the International Assessment of Adult Competencies (PIAAC) to provide an overview of AET participation, income, and technology-related problem-solving skills by sex and age groups in the four countries. Vickerstaff and van der Horst use data from five different organizations in the United Kingdom to examine attitudes of older workers about participation in training and the extent to which these attitudes result from self-imposed ageism. Taylor presents survey data from Australia that analyzes types of training women are undertaking, factors associated with participation in training, and the importance of employer support. Finally, Harrington and Cummins use PIAAC data to analyze age variations in AET participation and gender differences in employer sponsored training in Canada.

